# Upregulation of LncRNA Malat1 Induced Proliferation and Migration of Airway Smooth Muscle Cells *via* miR-150-eIF4E/Akt Signaling

**DOI:** 10.3389/fphys.2019.01337

**Published:** 2019-10-22

**Authors:** Li Lin, Qinghai Li, Wanming Hao, Yu Zhang, Long Zhao, Wei Han

**Affiliations:** ^1^Department of Pulmonary Medicine, Qingdao Municipal Hospital, School of Medicine, Qingdao University, Qingdao, China; ^2^Department of Ophthalmology, Qingdao Municipal Hospital, School of Medicine, Qingdao University, Qingdao, China; ^3^Department of Clinical Laboratory, Qingdao Municipal Hospital, School of Medicine, Qingdao University, Qingdao, China

**Keywords:** asthma, Malat1, miR-150, eIF4E, Akt signaling

## Abstract

The increased proliferation and migration of airway smooth muscle cells (ASMCs) are critical processes in the formation of airway remodeling in asthma. Long non-coding RNAs (lncRNAs) have emerged as key mediators of diverse physiological and pathological processes, and are involved in the pathogenesis of various diseases, including asthma. LncRNA Malat1 has been widely reported to regulate the proliferation and migration of multiple cell types and be involved in the pathogenesis of various human diseases. However, it remains unknown whether Malat1 regulates ASMC proliferation and migration. Here, we explored the function of Malat1 in ASMC proliferation and migration *in vitro* stimulated by platelet-derived growth factor BB (PDGF-BB), and the underlying molecular mechanism involved. The results showed that Malat1 was significantly upregulated in ASMCs treated with PDGF-BB, and knockdown of Malat1 effectively inhibited ASMC proliferation and migration induced by PDGF-BB. Our data also showed that miR-150 was a target of Malat1 in ASMCs, and inhibited PDGF-BB-induced ASMC proliferation and migration, whereas the inhibition effect was effectively reversed by Malat1 overexpression. Additionally, translation initiation factor 4E (eIF4E), an important regulator of Akt signaling, was identified to be a target of miR-150, and both eIF4E knockdown and Akt inhibitor GSK690693 inhibited PDGF-BB-induced ASMC proliferation and migration. Collectively, these data indicate that Malat1, as a competing endogenous RNA (ceRNA) for miR-150, derepresses eIF4E expression and activates Akt signaling, thereby being involved in PDGF-BB-induced ASMC proliferation and migration. These findings suggest that Malat1 knockdown may present a new target to limit airway remodeling in asthma.

## Introduction

Asthma is a common chronic respiratory disease, characterized by persistent airway inflammation, airway hyperresponsiveness, and airway remodeling, and affects 5% of adults and 10% of children with an increase in incidence (Bousquet et al., 2010; Poon and Hamid, 2016). Airway inflammation and hyperresponsiveness can be effectively managed by anti-inflammatory agents and bronchodilators, respectively, and airway remodeling remains intractable to existing treatments. In recent years, substantial research efforts have focused on clarifying the molecular mechanisms of airway remodeling. Undoubtedly, a deeper understanding of the molecular mechanism of airway remodeling will facilitate the development of the more effective therapy for asthma. Airway remodeling is characterized by subepithelial fibrosis, airway wall thickening, epithelial cell shedding, airway smooth muscle (ASM) hyperplasia and hypertrophy, angiogenesis, and mucus hypersecretion. Recently, increasing evidence suggests that abnormal proliferation and migration of ASM cells (ASMCs), the main structural component of the airway, are responsible for the change of ASM thickness and contribute to the progression of airway remodeling (Lazaar and Panettieri, 2005; Bentley and Hershenson, 2008; Prakash, 2013). Several inflammatory mediators play important roles in airway remodeling (Clarke et al., 2009; Finiasz et al., 2011). Among them, platelet-derived growth factor BB (PDGF-BB), significantly upregulated in asthmatic tissues, has been widely reported to be able to induce ASMC proliferation and migration and exacerbate the airway remodeling (Chanez et al., 1995; Yamashita et al., 2001; Zou et al., 2014; Liu et al., 2015; Zhang et al., 2016; Dai et al., 2018a). Thus, inhibition of PDGF-BB-induced ASMC proliferation and migration might represent a promising therapeutic option for asthma treatment.

In recent years, long non-coding RNAs (lncRNAs) have emerged as key mediators of diverse physiological and pathological processes. LncRNAs are non-coding RNA fragments comprising over 200 nucleotides that regulate gene expression *via* various mechanisms, including genomic imprinting, transcription, posttranscriptional processing, and chromatin modification (Ponting et al., 2009; Wang and Chang, 2011; Rinn and Chang, 2012). An extensive body of research has demonstrated the pivotal role of lncRNAs in the pathophysiology of asthma (Wang, 2017; Wang et al., 2017; Qi et al., 2018). Additionally, several recent studies indicate that lncRNAs could regulate ASMC proliferation and migration, and are involved in airway remodeling. For example, Zhang et al. (2016) reported that lncRNA BCYRN1 promotes rat ASMC proliferation and migration in asthma *via* upregulation of transient receptor potential 1; and Austin et al. (2017) identified lncRNA PVT1 as a novel mediator of the asthmatic phenotype in human ASM. LncRNA metastasis-associated lung adenocarcinoma transcript 1 (Malat1), a highly conserved nuclear lncRNA, is expressed at high level in most cells. Existing research suggests that Malat1 is involved in the pathogenesis of various human diseases, especially cancer. Malat1 has been termed an oncogene, which is upregulated in many cancers and promotes cancer initiation and progression (Gutschner et al., 2013; Wei and Niu, 2015). In addition, a number of recent studies point to the involvement of Malat1 in the pathogenesis of respiratory diseases. For example, Zhuo et al. (2017) reported that functional polymorphism of Malat1 contributes to pulmonary arterial hypertension susceptibility in Chinese people; Gutschner et al. (2012) demonstrated Malat1 as a critical regulator of the metastasis phenotype of lung cancer cells; Yan et al. (2017) reported that Malat1 modulates epithelial-mesenchymal transition in silica-induced pulmonary fibrosis *via* controlling miR-503/PI3K p85 signaling pathway; and Dai et al. (2018b) found that knockdown of Malat1 plays a protective role in the LPS-induced acute lung injury rat model. For Malat1 in asthma, in view that silencing of Malat1 profoundly impairs endothelial cell proliferation, Xue et al. (2017) elaborated that inhibition of Malat1 appears to be a promising approach to suppress airway epithelial cell proliferation, and reduce obstructive airway remodeling. However, in fact, the exact role of Malat1 in asthma has not been reported.

Here, we determined Malat1 expression in ASMCs treated with PDGF-BB, and explored the role of Malat1 in ASMC proliferation and migration induced by PDGF-BB and the underlying molecular mechanism involved.

## Materials and Methods

### Isolation and Culture of Airway Smooth Muscle Cells

ASMCs were prepared by the explant method from healthy segments of the main tracheas from three patients who underwent lung resection at the Qingdao Municipal Hospital. The study was approved by the Qingdao Municipal Hospital ethics committee, and the patients signed informed consent. Briefly, ASM bundles were isolated from surrounding tissues. Then the bundles were cut into 0.5-mm^3^ pieces, and placed in DMEM medium containing 20% fetal bovine serum (FBS; Gibco, Grand Island, NY, USA), 100 U/ml penicillin (Invitrogen, Carlsbad, CA, USA), and 100 μg/ml streptomycin (Invitrogen) in a humidified incubator at 37°C with 5% CO_2_. ASMCs were identified by morphological observation under an inverted light microscope (Olympus, Inc., Japan), and immunofluorescence staining for α-smooth muscle actin (α-SMA), a contractile phenotype marker protein. ASMCs were then cultured in DMEM medium supplemented with 10% FBS, 100 U/ml penicillin, and 100 μg/ml streptomycin. The medium was changed every 3 days until they formed a confluence and passaged using trypsin-EDTA (0.25% trypsin, 1 mM EDTA in HBSS; Gibco) solution. Cells at passages 4–6 were serum deprived for 24 h prior to treatment with 25 ng/ml of PDGF-BB.

### Immunofluorescence

Immunofluorescence was performed as previously described (Huo et al., 2018). Briefly, ASMCs were fixed with 4% paraformaldehyde for 15 min, and subsequently blocked with 5% BSA for 2 h. After incubating with α-SMA antibody at 4°C overnight, the cells were incubated with fluorescein isothiocyanate (FITC)-labeled secondary antibody for 1 h at 37°C, and 4′-6-diamidino-2-phenylindole (DAPI; Beyotime, Shanghai, China) was used to stain the nucleus. Images were captured using an inverted fluorescence microscope (Olympus, Inc.).

### Reverse Transcription-Polymerase Chain Reaction

Total RNA was extracted from cultured cells using TRIzol Reagent (Invitrogen) following the instructions of the manufacturer. For analysis of Malat1 and translation initiation factor 4E (eIF4E) expression, cDNA was synthesized by PrimeScript™ RT reagent kit (Takara, Dalian, China), and then added to Master Mix (Applied Biosystems, Carlsbad, CA) for subsequent PCR. The PCR products were separated by electrophoresis on a 2% agarose gel, and stained with ethidium bromide (EtBr; Applichem Inc., Cheshire, CT, USA). GAPDH was used as a reference. The band intensity was quantified with ImageJ 1.45 s software (National Institutes of Health, Bethesda, MD, USA). The primers used were as follows: Malat1, forward: 5′-AAAGCAAGGTCTCCCCACAAG-3′, and reverse: 5′-GGTCTGTGCTAGAT CAAAAGGCA-3′; eIF4E, forward: 5′-GGAAACCACCCCTACTCCTA-3′, and reverse: 5′-ATGGTTGTACAGAGCCCAAA-3′; GAPDH, forward: 5′-GGGCTCATCTGAAGGGTGGTGCTA-3′, and reverse: 5′-GTGGGGGAGACAGAAGGGAACAGA-3′. For analysis of miRNA expression, cDNA was synthesized by TaqMan MicroRNA Reverse Transcription kit (Applied Biosystems). Quantitative real-time PCR (qRT-PCR) was conducted on an ABI7300 System (Applied Biosystems) with SYBR Premix Ex Taq (Takara). U6 snRNA was used as a reference.

### Cell Transfection

Full-length Malat1 prepared by PCR was inserted into pcDNA3.1 vector (Sangon Biotech, Shanghai, China) respectively, and named as pcDNA-Malat1. The empty pcDNA3.1 vector was used as negative control, and named as pcDNA-NC. The miR-150 mimic, Malat1 small interfering RNA (si-Malat1), si-eIF4E, and their respective controls were synthesized by Shanghai GenePharma Co., Ltd. (Shanghai, China) as follows: miR-150 mimic, 5′-UCUCCCAACCCUUGUACCAGUG-3′; miR-NC, 5′-CAGUACUUUGUGUAGUACAA-3′; si-Malat1, 5′-GAGGUGUAAAGGGAUUUAUTT-3′; si-eIF4E, 5′-AAAGATAGTGATTGGTTAT-3′; and the negative siRNA, 5′-UUCUCCGAACGUGUCACGUTT-3′. ASMCs were grown on six-well plates, and once cells reached about 60% confluence, transfection reactions were performed by using Lipofectamine 2000 Reagent (Invitrogen) in accordance with the manufacturer’s instructions. After 48 h of transfection, cells were harvested for the following experiments.

### MTT Assay

Cell proliferation was assessed by MTT assay. In detail, cells at a density of 1 × 10^4^ cells/well were seeded on 96-well plates containing the medium with 25 ng/ml of PDGF-BB or not. After incubation at 37°C for 24 h, 20 μl of MTT (5 mg/ml) solution was added to each well, followed by additional incubation for 4 h at 37°C. Then the medium was removed and 200 μl of dimethyl sulfoxide (DMSO; Sigma Chemical Co.) was added to each well for solubilization of the MTT-formazan crystals. The absorption at 570 nm was read in a microplate reader (Dynatech, MR 7000, Billinghurst, UK). For Akt inhibitor study, ASMCs were grown in the culture medium containing 10 μM of Akt inhibitor GSK690693 (Selleck Chemicals, Houston, TX, USA) in the presence of PDGF-BB for 24 h.

### Transwell Assay

Cell migration was detected by Transwell chamber (24-well insert, 8-μm pore size; Corning Costar, Cambridge, MA, USA). Briefly, the cells (1 × 10^5^) resuspended in 200 μl of serum-free medium were added into the upper compartment of transwell plates. The lower chamber was filled with medium containing 20% FBS with or without 25 ng/ml of PDGF-BB. After incubation for 24 h, the non-migrated cells were removed from the upper membrane surface by cotton swabs, while the migrated cells on the lower surface of the filter were fixed in methanol (Sigma Chemical Co.), stained with 0.4% crystal violet (Shanghai Chem. Plant, Shanghai, China), and counted in five random optical fields. For Akt inhibitor study, 10 μM of GSK690693 was also added to the lower chamber.

### Western Blot Assay

Western blot assay was performed as previously described (Dai et al., 2018a,b). Primary antibodies for PCNA, MMP-9, and GAPDH were purchased from Santa Cruz Biotechnology (Santa Cruz, CA, USA). Primary antibodies for eIF4E, Akt, and phosphorylated Akt (p-Akt) were purchased from Abcam (Cambridge, MA, USA). Briefly, proteins were extracted from cultured cells using RIPA lysis buffer. Then protein were separated by 12% SDS-PAGE, and transferred to polyvinylidene difluoride (PVDF) membrane. The membranes were blocked with 5% skim milk at room temperature for 1 h, and then incubated overnight at 4°C with the primary antibodies. After incubation with HRP-conjugated secondary antibody for 1 h at room temperature, the blots were detected using an enhanced chemiluminescence kit (Pierce, Rockford, IL, USA). The integrated density of the blots was quantified by Image-Pro Plus 6.0 software (Media Cybernetics, Silver Spring, MD, USA).

### Luciferase Reporter Assay

The fragment 5′-aaaTGGAAAGATTAATTGGGAGtgg-3′ (position 1839) from Malat1 and the fragment 5′-tcaagcaatcgagatTTGGGAGc-3′ (position 52) from eIF4E 3′-UTR containing the predicted miR-150 target sites were chemically synthesized and individually inserted into the pMIR-luciferase reporter vector (Promega, Madison, WI, USA), and finally named as pMIR-Malat1-wt and pMIR-eIF4E-wt, respectively. The corresponding mutants were created by mutating the miR-150 seed region binding site (seed sequence binding fragment 5′-TGGAAAGATTAATTGGGAG-3′ from Malat1 changed to 5′-CCCTGGCTCCGGCCACCTC-3′, and seed sequence binding fragment 5′-TTGGGAG-3′ from eIF4E 3′-UTR changed to 5′-ACCCATA-3′), which were named as pMIR-Malat1-mut and pMIR-eIF4E-mut, respectively. ASMCs were cotransfected with pMIR-luciferase reporter vector containing Malat1 fragment or eIF4E 3′-UTR with wide type or mutant binding sites with miR-150, and mimic-NC or miR-150 mimic by using Lipofectamine 2000. Cells were harvested 48 h after transfection, and the luciferase activities were determined by using the Dual Luciferase Reporter Assay System (Promega).

### Statistical Analysis

All statistical tests were done on SPSS 17.0 statistical software (IBM, Chicago, IL, USA). All measurement data were presented as mean ± SD from three independent experiments (three independent experiments meaning use of cells from three different primary cultures). Student’s *t*-test was used to analyze the significance of mean values between two groups, and one-way ANOVA with *post hoc* Tukey’s test was used to compare the difference between multiple samples. Values of *p* less than 0.05 were considered to be statistically significant.

## Results

### Identification of Airway Smooth Muscle Cells

ASMCs were isolated from the healthy segments of the tracheas of the patients. To confirm successful isolation, we performed morphological observation under an inverted phase contrast microscope, and immunofluorescence staining for α-SMA. As shown in Supplementary Figures S1A,B, cells were fusiform that grew at distinctive rates with peaks and valleys, and expressed α-SMA, which confirmed the successful isolation of ASMCs.

### Malat1 Is Upregulated in Airway Smooth Muscle Cells Stimulated With Platelet-Derived Growth Factor-BB, and Malat1 Knockdown Inhibits Platelet-Derived Growth Factor-BB-Induced Airway Smooth Muscle Cell Proliferation and Migration

**Figure 1:**
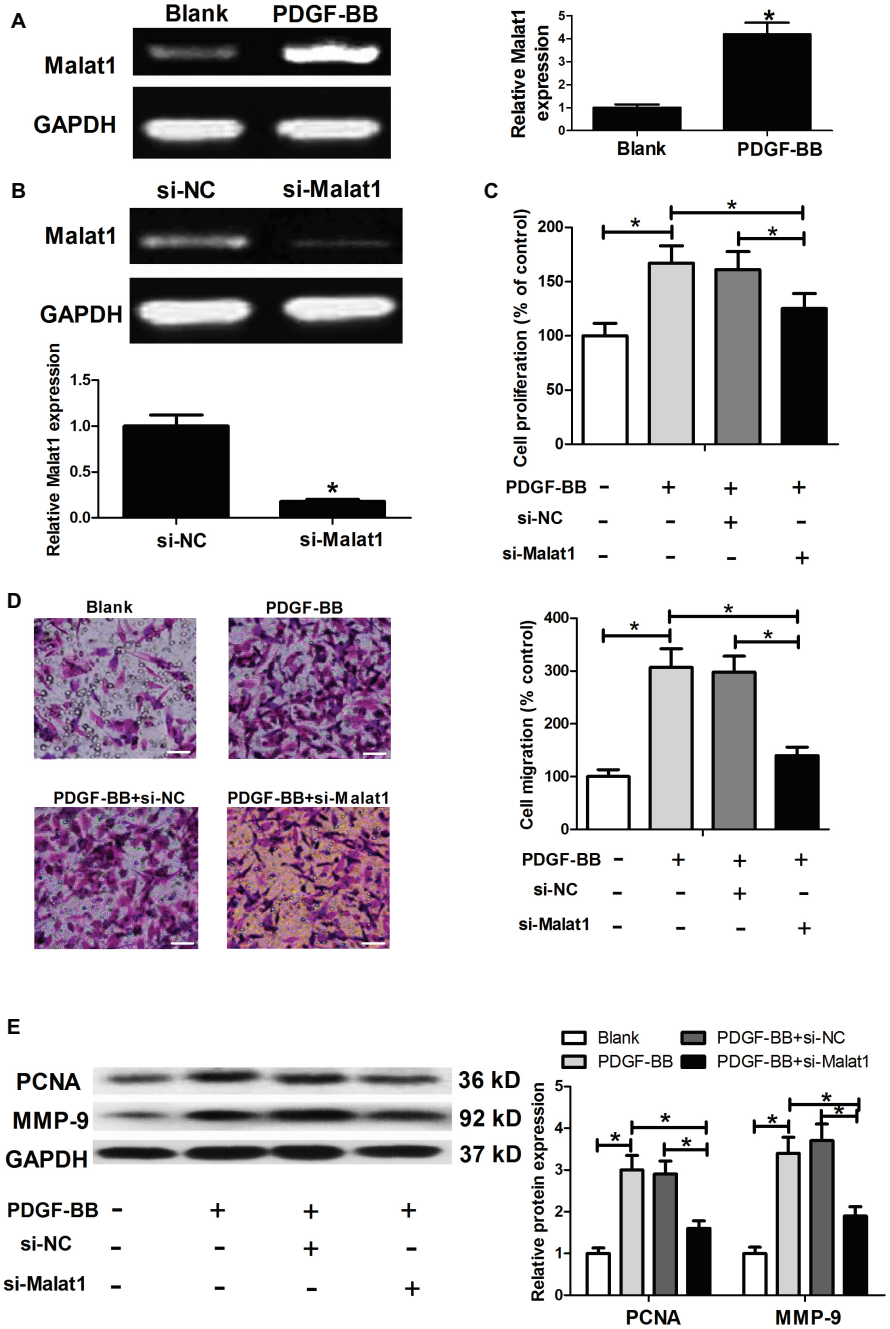
Malat1 is upregulated in ASMCs stimulated with PDGF-BB, and Malat1 knockdown inhibits PDGF-BB-induced ASMC proliferation and migration. **(A)** Malat1 expression in ASMCs treated with PDGF-BB determined by RT-PCR. **(B)** Malat1 expression in ASMCs transfected with si-Malat1 or si-NC determined by RT-PCR. **(C,D)** The effect of Malat1 on PDGF-BB-induced ASMC proliferation and migration assessed by MTT and Transwell assays, respectively (scale bar: 100 μm). **(E)** The effect of Malat1 on PCNA and MMP-9 expression in PDGF-BB-treated ASMCs determined by western blot assay. *p < 0.05.

To determine whether Malat1 expression was affected by PDGF-BB stimulation in ASMCs, ASMCs were cultured in complete medium containing 25 ng/ml of PDGF-BB or not for 24 h at 37°C, and then harvested for Malat1 expression detection by RT-PCR. As shown in Figure 1A, PDGF-BB stimulation significantly enhanced Malat1 expression in comparison with the blank group, implying that Malat1 might be involved in the regulation of PDGF-BB on the cell biological behaviors of ASMCs. For exploration of the role of Malat1 in ASMCs, we transfected ASMCs with si-Malat1 or si-NC, and generated Malat1 knockdown ASMCs confirmed by RT-PCR (Figure 1B). Then MTT and Transwell assays were performed to evaluate cell proliferation and migration. The results showed that PDGF-BB treatment significantly induced ASMC proliferation and migration in comparison with the blank group, and Malat1 knockdown significantly relieved the effect of PDGF-BB treatment on ASMC proliferation and migration (Figures 1C,D). In addition, western blot assay showed that PDGF-BB treatment significantly increased proliferation marker PCNA and migration-related protein MMP-9 expression compared to the blank group, and this effect was markedly reversed by Malat1 knockdown (Figure 1E). In addition, we also investigated the effect of Malat1 overexpression on the proliferation and migration of ASMCs untreated with PDGF. The results showed that Malat1 overexpression significantly promoted ASMC proliferation and migration (data not shown). Taken together, these data indicate that Malat1 plays an important role in PDGF-BB-induced ASMC proliferation and migration.

### Malat1 Targets miR-150

**Figure 2:**
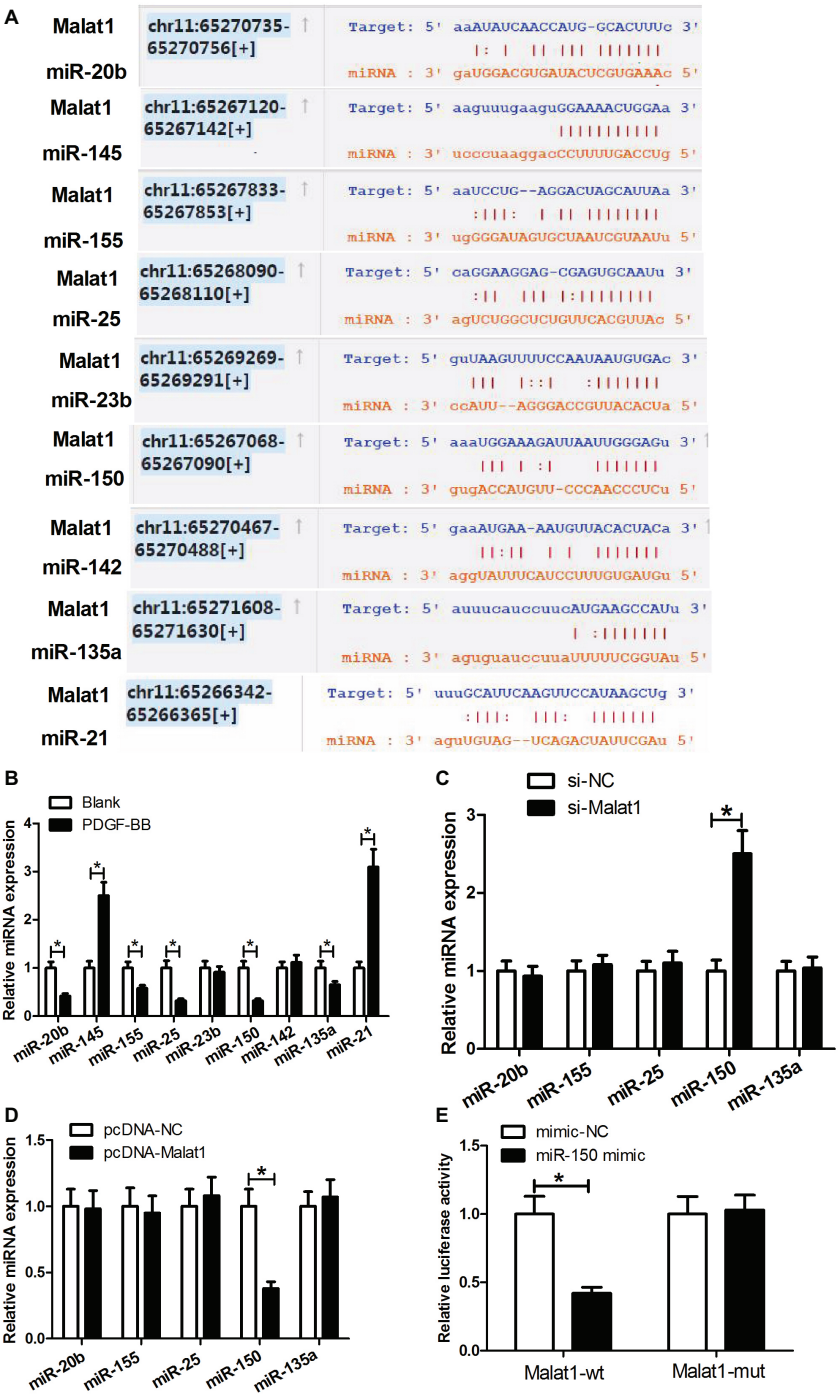
miR-150 is a target of Malat1. **(A)** Diagram of the putative binding sites of miR-20b, miR-145, miR-155, miR-25, miR-23b, miR-150, miR-142, miR-135a, and miR-21 in Malat1 sequences. **(B)** The effect of PDGF-BB stimulation on miR-20b, miR-145, miR-155, miR-25, miR-23b, miR-150, miR-142, miR-135a, and miR-21 expression in ASMCs determined by qRT-PCR. **(C,D)** The effect of Malat1 knockdown or overexpression on miR-20b, miR-155, miR-25, miR-150, and miR-135a expression in PDGF-BB-treated ASMCs determined by RT-PCR. **(E)** Luciferase reporter assays show that miR-150 mimic transfection significantly suppresses the luciferase activity of reporter vector containing Malat1-wt compared with the mimic-NC transfection, and has no influence on the luciferase activity of reporter vector containing Malat1-mut. *p < 0.05.

Growing evidence points that lncRNAs can work by competing with mRNAs for shared microRNAs (miRNAs), and making the derepression of the mRNAs. To explore whether Malat1 played its role through such a mechanism in ASMCs, we searched for the potential miRNAs that could interact with Malat1 *via* the online software starBase v3.0. From multitudinous candidate miRNAs, we selected miR-20b, miR-145, miR-155, miR-25, miR-23b, miR-150, miR-142, miR-135a, and miR-21 for further study since these miRNAs had been previously reported to be involved in asthma (Figure 2A). Then we determined the effect of PDGF-BB stimulation on the expression of these miRNAs in ASMCs by RT-PCR. As shown in Figure 2B, PDGF-BB treatment significantly enhanced miR-145 and miR-21 expression; reduced miR-20b, miR-155, miR-25, miR-150, and miR-135a expression; and had little impact on miR-23b and miR-142 expression in comparison with the blank group. Considering that lncRNA usually functions *via* a competing endogenous RNA (ceRNA) mechanism, we only focused on the reduced miRNAs miR-20b, miR-155, miR-25, miR-150, and miR-135a. Then we detected the effect of Malat1 expression alteration on the expression of these reduced miRNAs in PDGF-BB-treated ASMCs by RT-PCR. As shown in Figures 2C,D, Malat1 knockdown significantly enhanced miR-150 expression, and Malat1 overexpression obviously reduced miR-150 expression, while Malat1 expression alteration had no effect on the expression of miR-20b, miR-155, miR-25, and miR-135a. These results indicated that Malat1 only could modulate miR-150 expression. We further investigated whether there was a direct binding between Malat1 and miR-150 by performing luciferase reporter assay. As shown in Figure 2E, miR-150 mimic transfection significantly suppressed the luciferase activity of reporter vector containing Malat1-wt compared with the mimic-NC transfection, and had no influence on the luciferase activity of reporter vector containing Malat1-mut. Collectively, these data indicate that miR-150 is a target of Malat1, and Malat1 can directly modulate miR-150 expression.

### Malat1 Overexpression Markedly Reverses the Inhibitory Effect of miR-150 on Airway Smooth Muscle Cell Proliferation and Migration Induced by Platelet-Derived Growth Factor-BB

**Figure 3:**
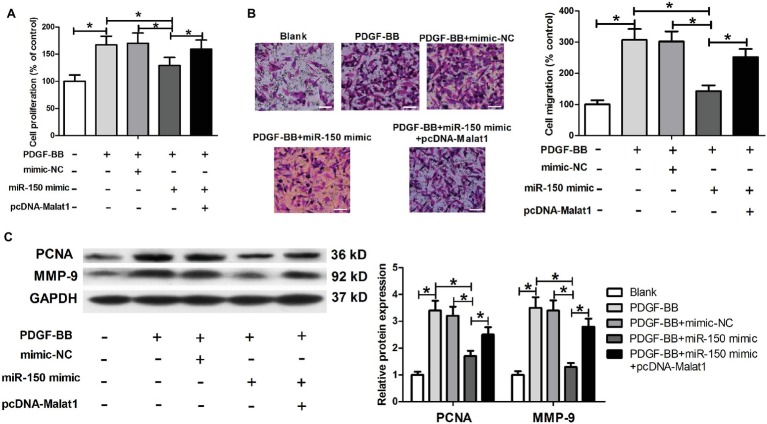
miR-150 inhibits PDGF-BB-induced ASMC proliferation and migration, and this inhibitory effect is markedly reversed by Malat1 overexpression. ASMCs are transfected with mimic-NC, miR-150 mimic, or miR-150 mimic + pcDNA-Malat1, and then used for proliferation and migration detection. **(A)** Cell proliferation assessed by MTT assay. **(B)** Cell migration detected by Transwell assay (scale bar: 100 μm). **(C)** PCNA and MMP-9 expression detected by western blot assay. *p < 0.05.

In view of the inhibitory effect of Malat1 on miR-150 expression, we suspected that the function of miR-150 could be suppressed by Malat1 in ASMCs. We transfected ASMCs with mimic-NC, miR-150 mimic, or miR-150 mimic + pcDNA-Malat1, and then conducted MTT, Transwell, and western blot assays. Results identified that miR-150 significantly inhibited ASMC proliferation and migration induced by PDGF-BB, while this inhibitory effect was significantly reversed by Malat1 overexpression (Figures 3A–C). These data indicate that Malat1 is involved in PDGF-BB-induced ASMC proliferation and migration *via* inhibition of miR-150 expression.

### eIF4E is a Direct Target of Malat1/miR-150 Signal Axis

**Figure 4:**
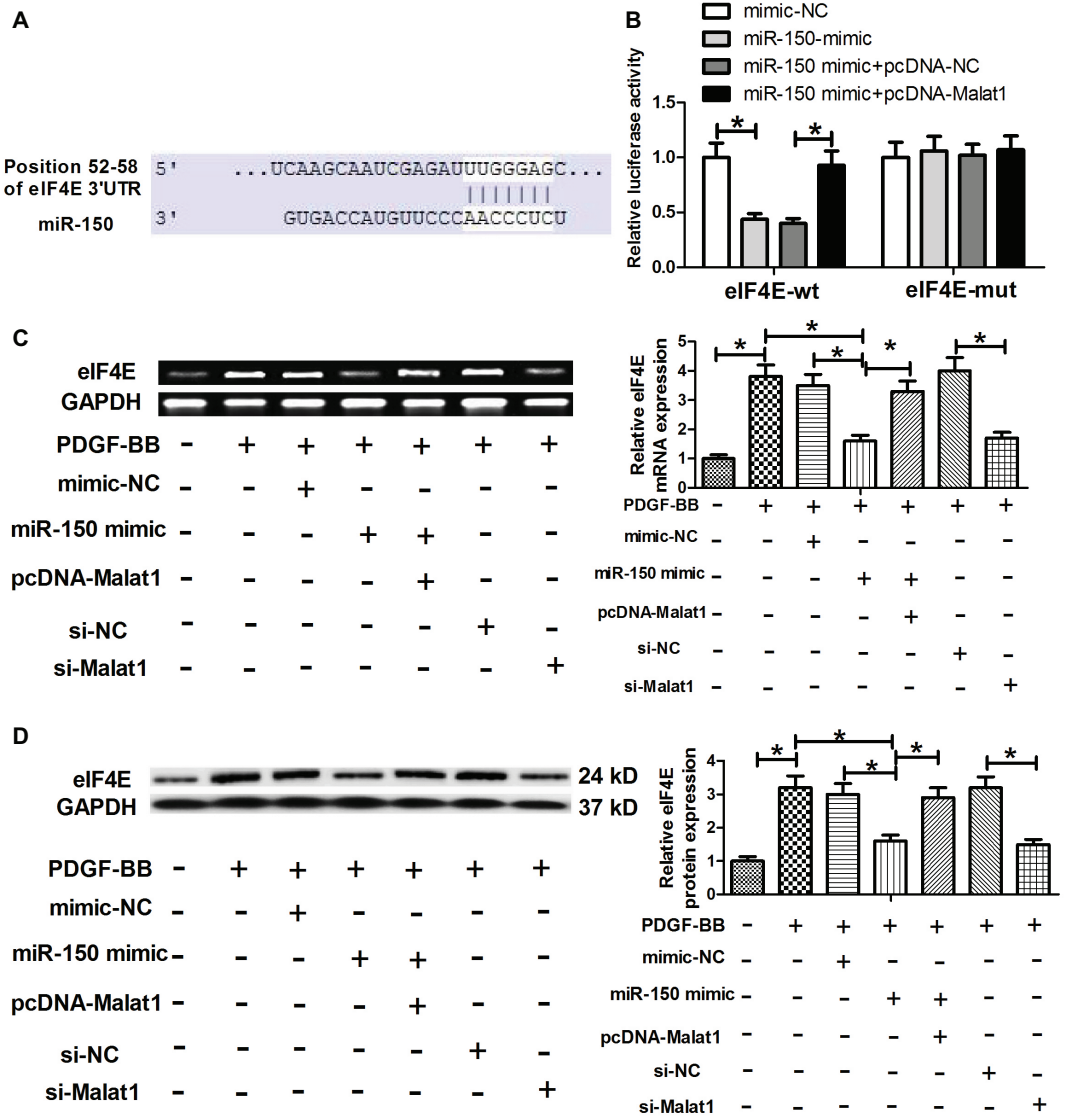
eIF4E is a direct target of Malat1/miR-150 signal axis. **(A)** Diagram of the miR-150 putative binding sites in eIF4E 3′-UTR. **(B)** Luciferase reporter assays show that miR-150 mimic transfection significantly reduces the luciferase activity of the reporter vector containing eIF4E-wt compared with mimic-NC transfection, whereas cotransfection with pcDNA-Malat1 and miR-150 mimic significantly relieves the inhibitory effect of miR-150 mimic transfection on luciferase activity. **(C,D)** eIF4E mRNA and protein expression in PDGF-BB-treated ASMCs transfected with mimic-NC, miR-150 mimic, miR-150 mimic + pcDNA-Malat1, si-NC, or si-Malat1 determined by RT-PCR and western blot, respectively. *p < 0.05.

To further explore the mechanism *via* which Malat1/miR-150 signal axis functioned in ASMCs, we performed target prediction *via* the online software TargetScan, and found that eIF4E was a potential target of miR-150 (Figure 4A). To verify that miR-150 could directly target the 3′-UTR of eIF4E, luciferase reporter assay was conducted. The results showed that miR-150 mimic transfection significantly reduced the luciferase activity of the reporter vector containing eIF4E-wt compared with mimic-NC transfection, whereas cotransfection with pcDNA-Malat1 and miR-150 mimic significantly relieved the inhibitory effect of miR-150 mimic transfection on luciferase activity, confirming that miR-150 could directly target the 3′-UTR of eIF4E (Figure 4B). Next, eIF4E mRNA and protein expression were determined in PDGF-BB-treated ASMCs transfected with mimic-NC, miR-150 mimic, miR-150 mimic + pcDNA-Malat1, si-NC, or si-Malat1. As shown in Figures 4C,D, PDGF-BB stimulation significantly enhanced eIF4E mRNA and protein expression, and the enhanced effects were significantly relieved by miR-150 overexpression or Malat1 knockdown, whereas the cotransfection with pcDNA-Malat1 and miR-150 mimic regained eIF4E mRNA and protein expression. Collectively, these data indicate that Malat1, as a ceRNA for miR-150, derepresses eIF4E mRNA.

### eIF4E Knockdown Inhibits Platelet-Derived Growth Factor-BB-Induced Airway Smooth Muscle Cell Proliferation and Migration

**Figure 5:**
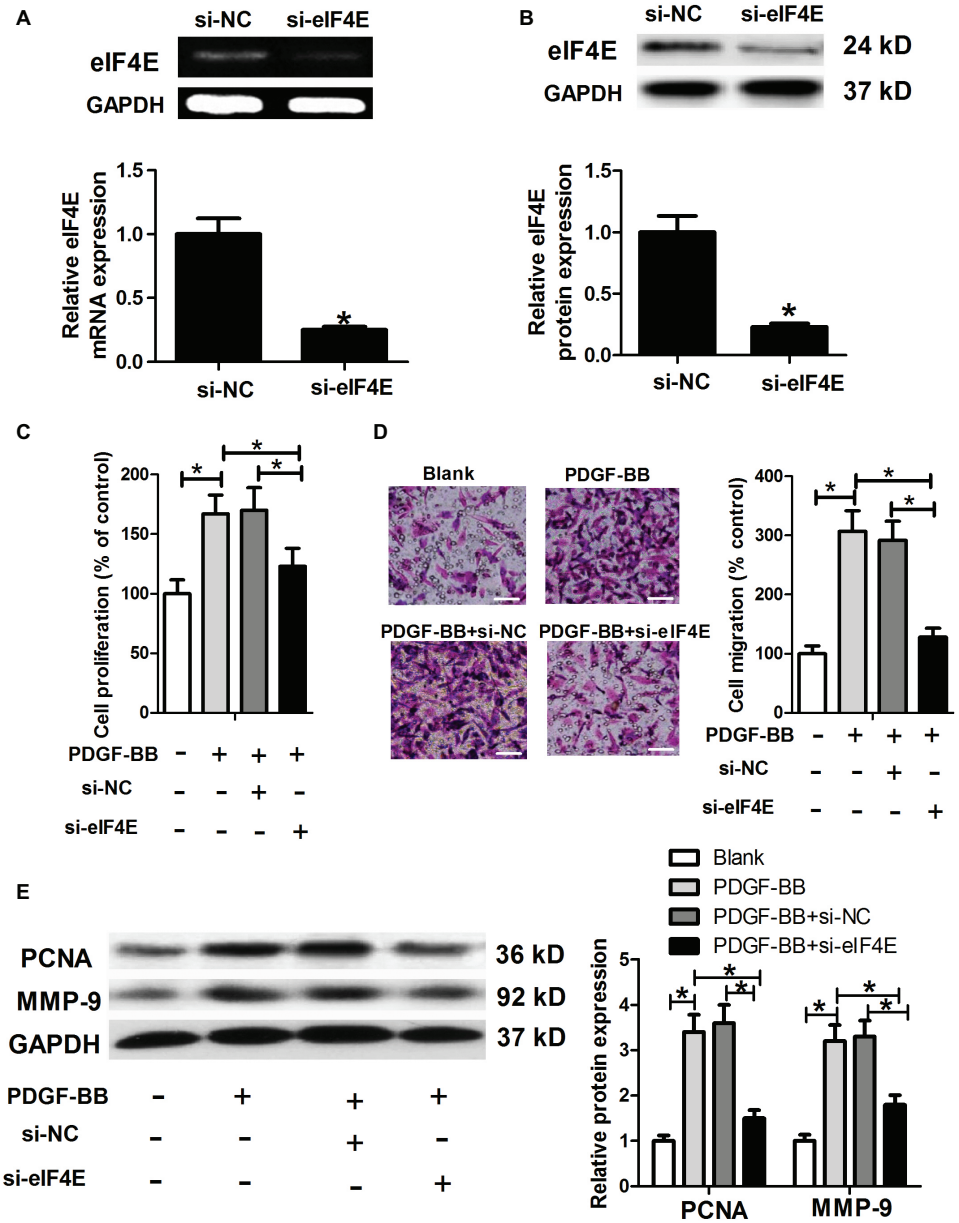
eIF4E knockdown inhibits PDGF-BB-induced ASMC proliferation and migration. ASMCs are transfected with si-NC or si-eIF4E, and then are harvested for the following detections. **(A,B)** eIF4E mRNA and protein expression detected by RT-PCR and western blot, respectively. **(C)** Cell proliferation determined by MTT assay. **(D)** Cell migration detected by Transwell assay (scale bar: 100 μm). **(E)** PCNA and MMP-9 expression determined by western blot assay. *p < 0.05.

To further investigate whether Malat1/miR-150 signal axis regulated PDGF-BB-induced ASMC proliferation and migration by targeting eIF4E, we generated eIF4E knockdown ASMCs by si-eIF4E transfection (Figures 5A,B), and then performed ASMC proliferation and migration detection. As shown in Figures 5C–E, eIF4E knockdown inhibited PDGF-BB-induced ASMC proliferation and migration.

### Malat1/miR-150/eIF4E Signal Axis Regulates the Akt Signaling in Platelet-Derived Growth Factor-BB-Treated Airway Smooth Muscle Cells

**Figure 6:**
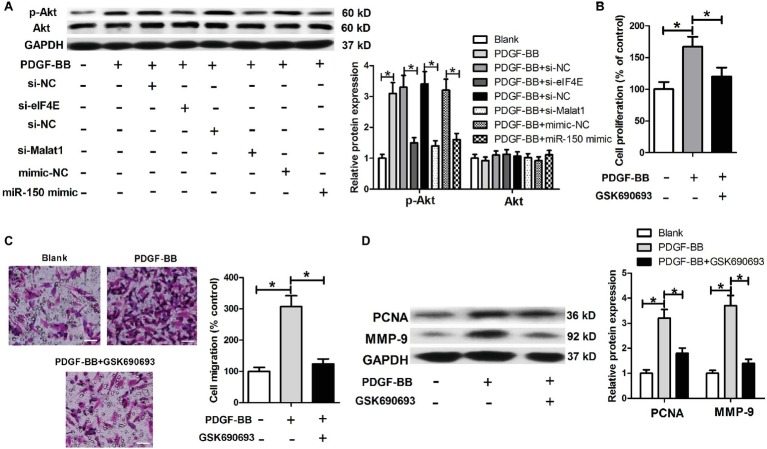
Malat1/miR-150/eIF4E signal axis regulates the Akt signaling in PDGF-BB-treated ASMCs. **(A)** Akt and p-Akt expression in PDGF-BB-treated ASMCs transfected with si-eIF4E, si-Malat1, miR-150 mimic, or their respective control. **(B–D)** The effects of GSK690693 on PDGF-BB-induced ASMC proliferation and migration determined by MTT, Transwell (scale bar: 100 μm), and western blot assays. *p < 0.05.

eIF4E has been widely reported to be functionally active *via* activation of the Akt signaling. We further investigated whether the Akt signaling was involved in the regulation of Malat1/miR-150/eIF4E signal axis on PDGF-BB-induced ASMC proliferation and migration. We first detected the effects of Malat1/miR-150/eIF4E signal axis on p-Akt and Akt expression in PDGF-BB-treated ASMCs, and the results showed that PDGF stimulation induced a significant increase in p-Akt expression, which was significantly downregulated by eIF4E knockdown, Malat1 knockdown, or miR-150 overexpression (Figure 6A). In addition, we detected the effect of Akt inhibitor GSK690693 on PDGF-BB-induced ASMC proliferation and migration by performing MTT, Transwell, and western blot assays. The results showed that GSK690693 inhibited PDGF-BB-induced ASMC proliferation and migration (Figures 6B–D). Collectively, these findings indicate that Malat1 is involved in PDGF-BB-induced ASMC proliferation and migration *via* interacting with miR-150 and activating eIF4E/Akt signaling.

## Discussion

The increased proliferation and migration of ASMCs are critical processes in the formation of airway remodeling in asthma (Lazaar and Panettieri, 2005; Bentley and Hershenson, 2008; Prakash, 2013). Diverse growth factors including PDGF, transforming growth factor, fibroblast growth factor, and epidermal growth factor, are main contributors to ASM remodeling, and their expression levels are directly correlated with asthma severity (Vignola et al., 1997; Puddicombe et al., 2000; Hoshino et al., 2001). Among these growth factors, PDGF family comprises five dimeric isoforms, and PDGF-BB, as it is a dimeric isoform, is secreted by inflammatory cells and airway epithelial cells in the asthmatic airways (Ohno et al., 1995; Shimizu et al., 2000; Fredriksson et al., 2004). Both *in vivo* and *in vitro* experiments have illustrated that PDGF-BB can drive the switch of ASMCs from a contractile phenotype to a proliferative, migratory, and synthetic phenotype, thereby causing ASMC proliferation and migration (Halayko et al., 2008; Ito et al., 2009; Hirota et al., 2011). Likewise, in this study, we also found that PDGF-BB stimulation significantly promoted ASMC proliferation and migration. In fact, PDGF-BB-induced proliferation and migration of ASMCs has been taken as a good model for studying asthmatic ASMC proliferation and migration *in vitro.*

Malat1 has been widely reported to be able to regulate the proliferation and migration of multiple cell types and involved in the pathogenesis of various human diseases. However, there is no report about the regulation of Malat1 on ASMC proliferation and migration in asthma. In this study, we found that Malat1 was significantly enhanced in ASMCs treated with PDGF-BB compared with the blank group, and Malat1 knockdown efficiently inhibited PDGF-BB-induced ASMC proliferation and migration, which was consistent with what Xue et al. (2017) demonstrated, that is, knockdown of Malat1 suppresses airway epithelial cell proliferation, and reduces obstructive airway remodeling. We further investigated the molecular mechanism by which Malat1 functioned.

miRNAs, a class of small non-coding RNAs comprising 19–25 nucleotides, inhibit gene expression at the posttranscriptional level by binding the 3′-UTR of target mRNA, and causing mRNA degradation, translation inhibition, or both. Substantial research has shown that miRNAs regulate many cellular processes, such as cell proliferation, differentiation, apoptosis, invasion, and migration, and are involved in the pathogenesis of diverse human diseases, including asthma.

In recent years, growing evidence points that lncRNAs can work by a ceRNA mechanism, where lncRNAs interact with miRNAs, causing the derepression of miRNA target genes. In fact, Malat1 has been widely reported to function *via* this mechanism. In this study, we hypothesized that Malat1 functioned in PDGF-BB-induced ASMC proliferation and migration *via* the ceRNA mechanism. To verify our hypothesis, we performed bioinformatics prediction by starBase v3.0 to identify the potential miRNAs that could interact with Malat1. From multitudinous candidate miRNAs, we selected nine miRNAs for further analysis since these miRNAs had been previously reported to be able to regulate the phenotype of asthmatic ASMCs. For example, miR-20b inhibits PDGF-induced human fetal ASMC proliferation by targeting STAT3; miR-155 inhibits IL-13-induced human ASMC proliferation and migration by targeting TGF-β-activated kinase 1/MAP3K7-binding protein 2; and miR-150 and miR-135a, upregulated by Schisandrin, inhibits ASMC proliferation and migration in asthmatic rats (Kuhn et al., 2010; Bartel et al., 2018; Shi et al., 2018). Next, we performed a series of experiments and found that in these miRNAs, Malat1 only could directly target miR-150, and regulate miR-150 expression. The inhibitory effect of miR-150 on PDGF-BB-induced ASMC proliferation and migration was markedly reversed by Malat1 overexpression. These data suggest that the inhibition of PDGF-BB-stimulated ASMC proliferation and migration induced by Malat1 knockdown requires the activity of miR-150.

eIF4E, an essential translation initiation factor, regulates gene expression through its mRNA export and translation functions. Increasing evidence suggests that high expression of eIF4E is related with oncogenic transformation in cell culture, tumor initiation and progression in mouse models, and poor prognosis in various human cancers (Graff and Zimmer, 2003). Recently, eIF4E has been reported to be required for ASMC hypertrophy in asthma, and inhibiting the phosphorylation of eIF4E may present a new therapeutic option to limit inflammation and remodeling in asthmatic airways (Zhou et al., 2005; Seidel et al., 2016). Here, we confirmed that eIF4E was a target of miR-150, and eIF4E knockdown inhibited PDGF-BB-induced ASMC proliferation and migration. eIF4E has been widely reported to function *via* increasing the phosphorylation of Akt, and activating Akt survival signaling (Nathan et al., 2004; Culjkovic et al., 2008; Tan et al., 2008). In addition, previous studies have also showed that PDGF-BB treatment promotes ASMC proliferation and migration *via* activation of several signaling pathways, including Akt pathway (Liu et al., 2015). These findings tempted us to speculate that Malat1/miR-150/eIF4E signal axis functioned in PDGF-BB-treated cells *via* regulation of Akt signaling. A series of experiments were performed, and the results confirmed our speculation.

## Conclusions

In conclusion, our study first uncovers that PDGF-BB stimulation upregulates Malat1 expression in ASMCs, and knockdown of Malat1 inhibits PDGF-BB-induced ASMC proliferation and migration *via* interaction with miR-150 and blockade of eIF4E/Akt signaling. These findings suggest that Malat1 knockdown may present a new target to limit airway remodeling in asthma.

## Data Availability Statement

All datasets generated for this study are included in the manuscript/Supplementary Files.

## Ethics Statement

ASMCs were prepared by the explant method from healthy segments of the main tracheas from three patients who underwent lung resection at the Qingdao Municipal Hospital. The study was approved by the Qingdao Municipal Hospital ethics committee, and the patients signed informed consent.

## Author Contributions

LZ and WHan designed the experiments. LL, QL, and WHao carried out the experiments and edited the manuscript. YZ carried out statistical analyses and edited figures. All authors read and approved the final manuscript.

### Conflict of Interest

The authors declare that the research was conducted in the absence of any commercial or financial relationships that could be construed as a potential conflict of interest.

## Supplementary Material

SUPPLEMENTARY FIGURE S1 Identification of airway smooth muscle cells (ASMCs). **(A)** ASMCs are fusiform that grow at distinctive rates with peaks and valleys under an inverted phase contrast microscope (scale bar: 50 μm). **(B)** Immunofluorescence staining shows positive expression of α-SMA (green) in ASMCs (scale bar: 20 μm).

## Abbreviations

ASM: Airway smooth muscle
ASMC: Airway smooth muscle cell
ceRNA: Competing endogenous RNA
lncRNA: Long non-coding RNA
PDGF-BB: Platelet-derived growth factor BB
RT-PCR: Reverse transcription-polymerase chain reaction
α-SMA: α-Smooth muscle actin
eIF4E: Translation initiation factor 4E.
