# Supplementary figures and images for: Upregulation of LncRNA Malat1 Induced Proliferation and Migration of Airway Smooth Muscle Cells via miR-150-eIF4E/Akt Signaling
Source: Front Physiol. 2019 Oct 22;10:1337. doi: 10.3389/fphys.2019.01337 (PMC6817469; doi:10.3389/fphys.2019.01337)

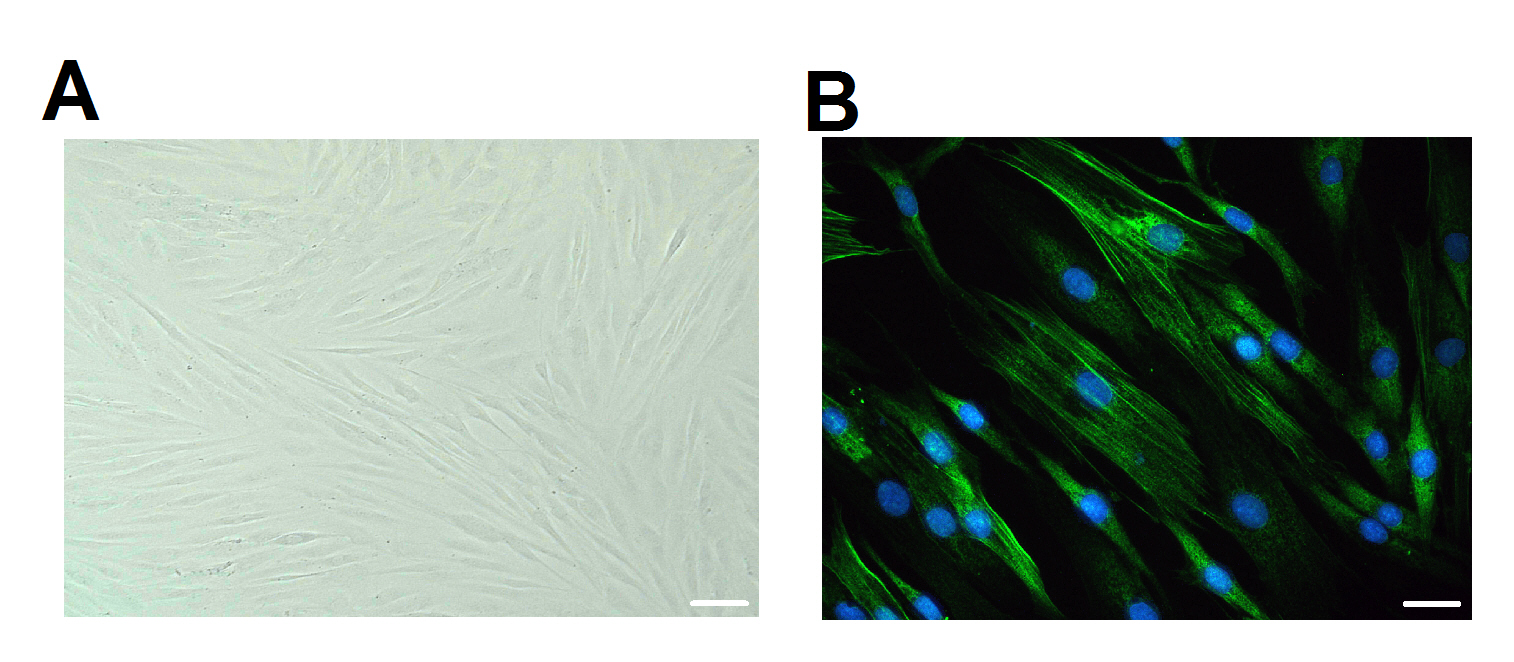

Supplement: SUPPLEMENTARY FIGURE S1 — Identification of airway smooth muscle cells (ASMCs). (A) ASMCs are fusiform that grow at distinctive rates with peaks and valleys under an inverted phase contrast microscope (scale bar: 50 μm). (B) Immunofluorescence staining shows positive expression of α-SMA (green) in ASMCs (scale bar: 20 μm). [file Image_1.JPEG]
